# Vitamin D Status in Patients with Systemic Lupus Erythematosus in Serbia: Correlation with Disease Activity and Clinical Manifestations

**DOI:** 10.3889/oamjms.2015.052

**Published:** 2015-05-04

**Authors:** Rada Miskovic, Aleksandra Plavsic, Sanvila Raskovic, Zikica Jovicic, Jasna Bolpacic

**Affiliations:** *Clinical Center of Serbia, Clinic for Allergology and Immunology, Belgrade, Serbia*

**Keywords:** SLE, vitamin D, disease activity, glucocorticoids, vitamin D supplements

## Abstract

**BACKGROUND:**

Numerous studies indicate potential role of vitamin D as an important factor in the development of many autoimmune diseases including systemic lupus erythematosus (SLE). Patients with SLE are especially prone to the development of vitamin D deficiency due to the nature of their illness.

**AIM:**

The aims of our study were to determine the prevalence of vitamin D insufficiency and deficiency in patients with SLE in Serbia, to identify clinical variables associated with vitamin D status and to examine the impact of vitamin D status on disease activity and presence of specific lupus autoantibodies.

**MATERIAL AND METHODS:**

The study included 46 patients with SLE. Serum 25(OH)D concentration was measured by electrohemiluminiscent immunoassay.

**RESULTS:**

The mean serum concentration of 25(OH)D was 11.9 ± 7.3 ng/ml. The prevalence of insufficiency was 32.6%, while the prevalence of deficiency was 67.4%. There was no association between vitamin D status and photosensitivity, skin lesions, arthritis and lupus nephritis. Vitamin D status was not associated with the presence of specific autoantibodies. There was no correlation between disease activity assessed by SLEDAI scale with the concentration of 25(OH)D. Patients who used vitamin D supplements and calcium did not have a significantly higher concentration of 25(OH)D.

**CONCLUSION:**

In conclusion, vitamin D deficiency is common in patients with SLE.

## Introduction

Vitamin D is a liposoluble hormone with an essential role in the metabolism of calcium and phosphorus and bone mineralization. The main source of vitamin D is endogenous synthesis in the skin under the action of ultraviolet B rays, after which it gradually undergoes the process of hydroxylation, first in the liver when 25-hydroxy-vitamin D (25(OH)D) is produced, and then in the kidneys to make the active form of vitamin D - 1,25-dihydroxy-vitamin D (1,25(OH)2D). Smaller amounts of vitamin D come from food and supplements [1].

The role of vitamin D in the immune system was discovered in the 1980s when it was shown that antigen-presenting cells and lymphocytes express receptor for vitamin D (VDR). Further studies have established that many cells of the immune system have enzymes responsible for the metabolism of vitamin D which enables them to produce the active form of vitamin D in the microenvironment of lymph tissues. The effect of vitamin D on dendritic cells (DCs) is particulary important in autoimmunity. Vitamin D inhibits differentiation and promotes the formation of tolerogenic DCs with immature phenotype. It suppresses the differentiation of Th1 lymphocytes, stimulates T regulatory cells, reduces the production of autoantibodies and release of inflammatory mediators [2-4]. There are evidence that vitamin D suppresses the expression of “interferon signature” as an important mechanism in the SLE pathogenesis [5]. Studies on several animal models of lupus showed that administration of vitamin D can lead to the improvement of various disease manifestations [6].

Numerous studies have found a high prevalence of vitamin D deficiency in patients with SLE. Some of the possible reasons for this are photosensitivity and application of sunscreen with a high sun protection factor, kidney damage, chronic glucocorticoids therapy, the use of the antimalarials [7]. Many authors point to the association between higher disease activity and vitamin D deficiency [8, 9]. However, in some studies, this association was not found [10].

Our study was designed as a cross sectional study with the following objectives: (1) to determine the prevalence of vitamin D deficiency and insufficiency in SLE patients; (2) to examine the association between selected clinical manifestations of SLE with the vitamin D status; (3) to examine the association between vitamin D status with the presence of specific lupus autoantibodies in the serum of SLE patients; and (4) to examine the association between SLE activity and vitamin D status.

## Materials and Methods

### Subjects and variables

The study included 46 consecutive SLE inpatients at the Clinic for Allergology and Immunology, Clinical Center of Serbia during the period from May to October 2011. All patients fulfilled at least 4 of 11 American College of Rheumatology (ACR) criteria from 1997 for the diagnosis of SLE. Exclusion criteria were: pregnancy and lactation period, elevated serum creatinine and diagnosis of malignant diseases or suspected malignancy. The study was approved by the Ethical committee of the Clinical Center of Serbia.

The following variables were recorded: age, sex, duration of SLE, photosensitivity, skin changes, active arthritis, and activity of lupus nephritis and use of glucocorticoids (GC), antimalarials and vitamin D and calcium supplements. All patients underwent following laboratory analyzes: complete blood count with differential leukocyte formula, standard biochemical analysis, concentration of parathyroid hormone, urinalysis, 24h-proteinuria, creatinine clearance, C3 and C4 concentration, presence of the following autoantibodies: ANA, anti-ds-DNA, anti-SSA, anticardiolipin IgG and IgM Ab (aCL). Disease activity was evaluated using SLEDAI (SLE Disease Activity Index) scale.

### Determination of vitamin D status

Vitamin D status was determined by measuring serum 25(OH)D concentration, which is the main circulating form of vitamin D. We used cut-off values recommended by the laboratory where the analysis was preformed: insufficiency was defined as serum 25(OH)D concentration less than or equal 30ng/ml and greater than 15ng/ml, while 25(OH)D concentration less than or equal to 15 ng/ml was defined as a deficiency. Blood samples from all patients were taken during summer period (May to October). The 25(OH)D concentration was measured using ECLIA (electrochemiluminescent immunoassay) on the Roche Cobas E601 apparatus.

### Statistical analysis

The data collected were processed using standard statistical methods using statistical software SPSS 14:00 for Windows (SPSS Inc., Chicago, IL, USA).We used following statistical methods: descriptive statistics, methods for testing the significance of differences (t-test, chi-square test, ANOVA) and methods for assessing correlation (Pearson and Spearman correlation). Prevalence of vitamin D deficiency and insufficiency was calculated as the ratio between the number of patients with a deficiency or insufficiency and the total number of subjects. All patients had reduced serum 25(OH)D concentration, therefore we compared patients with vitamin D insufficiency to patients with vitamin D deficiency.

## Results

### Description of the study group

Forty-six patients with a mean age of 45.4 ± 12.5 (21-68) years were included in the study. There were 40 (86.9%) female and 6 (13.1%) male patients. Mean disease duration was 6.15 (0-29) years. Table 1 shows main clinical, laboratory and therapeutic characteristics of the study group.

**Table 1:** Clinical, laboratory and therapeutic characteristics of the study group

Variable	Value
Photosensitivity (n /%)	27 (58.7%)
Skin lesions (n/%)	9 (19.6%)
Arthritis (n/%)	15 (32.6%)
Lupus nephritis (n/%)	22 (47.8%)
Hematologic manifestations (n/%)	14 (30.4%)
Serositis (n/%)	3 (6.5%)
Neurolupus (n/%)	5 (10.9%)
Serum calcium (mmol/l, range)	2.23 (1.93-2.58)
Parathyroid hormone (pg/ml, range)	56 (17-141)
Creatinin clearance (ml/min, range)	75.1 (28.08-136.96)
ANA (n, %)	36 (78.3%)
anti-ds-DNK Ab (n, %)	11 (24.0%)
anti-SSA Ab (n, %)	18 (39.1%)
aCL IgG (n, %)	6 (13.0%)
aCL IgM (n, %)	10 (21.7%)
SLEDAI 0 (n,%)	10 (21.7%)
SLEDAI 1-7 (n,%)	21 (45.7%)
SLEDAI ≥8	15 (32.6%)
Current GC therapy (n, %)	41 (89.%)
Average prednisolone dose (g, range)	0.015 (0-0.1)
Average duration of GC therapy (y, range)	6.15 (0-29)
Current antimalarials (n, %)	9 (19.6%)
Current vitamin D and calcium supplements (n,%)	22 (47.8%)

### Prevalence of vitamin D deficiency and insufficiency

**Figure 1:**
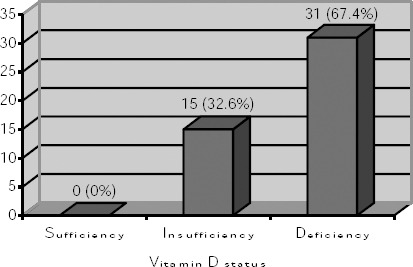
*Distribution of patients according to vitamin D status*.

Mean serum 25(OH)D concentration was 11.9 ± 7.3 ng/ml (0-25.7 ng/ml). All patients had reduced serum 25(OH)D concentration. Figure 1 shows distribution of patients according to vitamin D status.

The average 25(OH)D concentration in the insufficiency group was 20.8 ± 3.2 ng/ml, and in the deficiency group we measured 7.5 ± 3.9 ng/ml. There was a statistically significant negative correlation between serum 25(OH)D and PTH serum concentration (r = - 0.434, p = 0.003).

### Characteristics of patients according to the vitamin D status

Patients in the deficiency group did not differ significantly with respect to age (t = 0.889, p = 0.378), SLE duration (t = 1.73, p = 0.09) and gender (χ² = 0.002, p = 0.964) from those in the insufficiency group. Table 2 shows distribution of selected demographic, clinical and laboratory variables according to the vitamin D status.

**Table 2:** Distribution of demographic, clinical and laboratory variables according to the vitamin D status

Variable	Insufficiency	Deficiency
Age	43.1 ± 13.85	46.6 ± 11.91
Female gender	13/15 (86.7%)	27/31 (87.1%)
SLE duration	8.3 ± 7.62	5.1 ± 5.04
Photosensitivity	8/15 (53.3%)	19/31 (61.3%)
Skin lesions	3/15 (20.0%)	6/31 (19.4%)
Arthritis	6/15 (40.0%)	9/31 (29.0%)
Lupus nephritis	5/15 (33.3%)	17/31 (54.8%)
anti-ds-DNK Ab	2/15 (13.4%)	9/31 (29.0%)
anti-SSA Ab	4/15 (26.7%)	14/31 (45.2%)
SLEDAI 0	2/15 (13.4%)	8/31 (25.8%)
SLEDAI 1-7	8/15 (53.3%)	13/31 (41.9%)
SLEDAI ≥ 8	5/15 (33.3%)	10/31 (32.2%)
Current prednisolone dose in g	0.0127	0.0165
Duration of GC therapy (years)	8.3	5.1
Antimalarials	2/15 (13.4%)	7/31 (22.6%)
Vitamin D and calcium supplements	9/15 (60%)	13/31 (41.9%)

### Vitamin D and clinical manifestations of SLE

We examined the association of specific clinical manifestations with vitamin D status. There was no statistically significant difference in the incidence of photosensitivity (χ² = 0.264, p = 0.607), skin lesions (χ² = 0.003, p = 0.959) and arthritis (χ² = 0.553, p = 0.457) between deficiency and insufficiency group. There was a higher incidence of lupus nephritis in the vitamin D deficiency group (54.8% vs 33.3%), but it was not statistically significant (χ² = 1.874, p = 0.171).

### Vitamin D and presence of specific lupus autoantibodies

We investigated the influence of 25(OH)D concentration on the presence of specific lupus autoantibodies. None of tested autoantibodies showed a statistically significant association with vitamin D status (ANA - χ² = 0.924, p = 0.338; anti-ds-DNA At - χ² = 1.369, p = 0.241; anti-SSA At - χ² = 1.452, p = 0228; aCL IgG - χ² = 0.95, p = 0.329; aCL IgM class - χ² = 0.318, p = 0.572). Patients who had positive anti-ds-DNA and anti-SSA antibodies had lower mean 25(OH)D concentration, but this difference did not reach statistical significance (anti-ds-DNA At - t = 1.482, p = 0.145; anti-SSA At - t = 1.015, p = 0.316).

### Vitamin D and SLE activity

There was no correlation between 25(OH)D concentration and SLEDAI score (Pearson correlation coefficient 0.194, p = 0.195). Comparing the average 25(OH)D concentration by groups of SLEDAI score, there was no statistically significant difference between these groups (ANOVA, F = 1.406, p = 0.256). Mean SLEDAI score in the insufficiency group was not statistically significant different from the score in the vitamin D deficiency group (6.6 ± 7.34 vs 5.2 ± 4.61, t = 0.814, p = 0.42).

### Vitamin D and drugs use

Most of our patients used GC (41/46, 89.1%). The 25(OH)D concentration did not correlate to the current prednisolone dose (Spearman correlation coefficient of -0.116, p = 0.442) or to the duration of GC therapy (Spearman correlation coefficient 0.184, p = 0.221). There were no statistically significant differences between vitamin D deficiency and insufficiency group in relation to the current prednisolone dose (p = 0.45) and duration of GC therapy (p = 0.10).

According to the incidence of antimalarial use, there was no statistically significant difference between the two groups (χ² = 0.549, p = 0.458). The average 25(OH)D concentration in patients who used antimalarials was 10.1 ng/ml, which was less than in those who did not used them in therapy (12.3 ng/ml), but this was not statistically significant (t = 0.802, p = 0.427).

Almost half of the patients (47.8%) used vitamin D (400-800 IU cholecalciferol) and calcium supplements. Those who used supplements did not have significantly higher serum 25(OH)D concentration (12.7 vs. 11.67 ng/ml, t = 0.185, p = 0.854).

## Discussion

Patients with SLE have multiple risk factors for vitamin D deficiency. However, data on the prevalence of vitamin D deficiency in SLE show significant variation and range from 8% to 98% [11-17]. Our study showed decreased serum 25(OH)D concentration in all patients. Prevalence of vitamin D insufficiency and deficiency were 32.6% and 67.4%, respectfully. Many other studies found high prevalence of reduced vitamin D concentration in SLE patients [10, 11, 15, 18, 19]. In the study of Ruiz-Irastroza and al., 75% of patients had 25(OH)D less than 30 ng/ml and 15% had less than 10 ng/ml [18]. The study included 92 patients mostly with a mild form of disease, whereby 48% of them didn’t use GC in therapy at the time of determination of vitamin D status. Canadian study obtained similar data: 66.7% of SLE patients had 25(OH)D concentration less than 80 nmol/l (32 ng/ml), and 17.9% less than 40 nmol/l (16 ng/ml) [20]. In a study published in 2011, which included 177 patients with SLE treated at a single center in Hungary, 81.9% had 25(OH)D concentration less than 30 ng/ml [15]. High prevalence of low vitamin D concentration is found in a study from Saudi Arabia where 98.8% of patients had suboptimal vitamin D level. Study included 165 SLE inpatients who were compared with 214 healthy subjects in control group, where the prevalence of vitamin D deficiency was 55% [16]. However, several studies have showed low prevalence of vitamin D deficiency. Bultink and al. found vitamin D deficiency in only 8% of 107 SLE subjects [12]. Differences in study design, characteristics of study groups and reference values of 25(OH)D make direct comparison between studies very difficult.

Mean serum concentration of 25(OH)D in our group of patients was 11.9 ± 7.3 ng/ml (0-25.7 ng/ml). These results are similar to those from the group from Shanghai (11.5ng/ml) who showed significantly lower concentration of 25(OH)D in SLE patients than in those with rheumatoid arthritis (54.6 ng/ml) and control group (59.2 ng/ml) [21]. In Israeli study from 2008, serum 25(OH)D concentration was 11.9 ng/ml in SLE patients, which was significantly lower than in patients with other autoimmune diseases [10]. On the other hand, Kamen and al. measured mean serum 25(OH)D concentration of 21.6 ng/ml in SLE patients in a study from 2006 [11].

Almost half of our patients (47.8%) used vitamin D and calcium supplements. Daily dose of cholecalciferol in supplements ranged from 400-800 IU. We found no significant difference in 25(OH)D concentration among patients who used supplements and those who did not. Similar results were obtained in other studies and it seems that these doses of supplements do not provide complete protection from low vitamin D level and that these patients are also at risk for vitamin D deficiency [14, 18, 20, 22]. The contributing factor could be also a lack of compliance among patients. These results suggest that patients with SLE may require higher supplementation dose of vitamin D.

We found no difference in 25(OH)D concentration between patients who used antimalarials and those who did not, but the value of this finding is diminished by the fact that only 19.5% of subjects used antimalarials in therapy. Antimalarials affect metabolism of vitamin D by inhibiting 1-α-hydroxylation of 25(OH)D, thereby reducing serum concentration of active form of vitamin D [23]. This feature of antimalarials is the basis for their use in the treatment of 1,25(OH)2D-dependent hypercalcemia in granulomatous disease [24]. However, it is not completely understood how antimalarials affect 25(OH)D concentration. Huisman and al. found lower 1,25(OH)2D concentration in SLE patients treated with hydroxychloroquine and no significant difference in 25(OH)D concentration among patients who use hydroxychloroquine and those who did not [14]. On the other hand, Ruiz-Irastroza and al. found higher 25(OH)D concentration in SEL patients who were treated with hydroxychloroquine [18].

Long-term GC therapy affects metabolism of vitamin D. Animal studies have showed that high doses GCs stimulate activity of 24-hydroxylase and moderately decrease the activity of 1-alpha-hydroxylase in the kidneys. However, several studies addressing this issue have found both reduced and normal 25(OH)D concentration in patients with chronic GC therapy [20, 25-27]. In our study, 25(OH)D concentration did not correlate either to the current GC dose, nor to the length of GC therapy. It should be kept in mind that the majority of our subjects used moderate to high GC doses and all of them had reduced serum 25(OH)D concentration.

A link between vitamin D deficiency and certain clinical manifestations of SLE has been suggested in several studies. Kamen and al. found that photosensitivity and renal disease are, regardeless of race, strong predictors of critically low vitamin D level (less than 10 ng/ml) and in the Caucasians group the association between discoid rash and lower vitamin D level was found [11]. A study of SLE patients in Hungary found that those with pericarditis, neuropsychiatric manifestations and deep venous thrombosis had significantly lower vitamin D level [28]. In our study, we did not find an association between vitamin D status and skin lesions, photosensitivity, arthritis, and lupus nephritis. There was a higher prevalence of vitamin D deficiency in a group of patients with lupus nephritis, but it was not statistically significant (54.8% vs. 33.3%). Creatinine clearance did not correlate with 25(OH)D concentration. It is possible that impaired renal function leads to accumulation of 25(OH)D which overcomes loss of function of 1-α-hydroxylase in the kidneys.

Mean duration of SLE in our patients with vitamin D deficiency was 5.1 ± 5.0 years, which was less than in vitamin D insufficiency group where mean disease duration was 8.3 ± 7.6 years. This difference was not statistically significant. Many other studies did not find an association between disease duration and vitamin D status [18, 20]. It seems that vitamin D status is determined more by the way that disease is clinically manifested and by applied therapy, rather than disease duration.

A larger number of clinical studies dealt with the relationship between vitamin D status and SLE activity [8-10, 18, 29]. A study on 165 SLE patients in three centers showed significantly greater disease activity in patients with severe vitamin D deficiency [8]. A similar result was obtained in a group of 46 patients from northern and southern Europe [9]. On the other hand, several other studies did not find this association [10, 18, 21, 29]. Korean authors did not find correlation between serum 25(OH)D concentration and SLEDAI score and anti-dsDNA Ab in a study from 2010 which involved 104 SLE patients [29]. In our study, SLEDAI score did not differ significantly depending on the vitamin D status. Possible reasons for this result are relatively small number of subjects and the fact that majority of subjects had active disease at the time vitamin D status was determined. Also, all patients had reduced serum 25(OH)D concentration, so we couldn’t compare patients with vitamin D deficiency or insufficiency to patients who have sufficent vitamin D.

Basic studies have shown that vitamin D suppresses Th-1 immune response, facilitates induction of Foxp3+ regulatory T cells, interruptes the process of B lymphocytes differentiation and inhibits the production of immunoglobulins and autoantibodies [30-32]. One study demonstrated statistically significant negative correlation between vitamin D level and the presence of IgG anticardiolipin antibodies in patients with deep venous thrombosis [28]. The same study found statistically significantly higher anti-ds-DNA antibodies titer in a group of patients with vitamin D deficiency and insufficiency compared to patients who were vitamin D sufficient. Our results did not show any association between vitamin D deficiency and the presence of tested autoantibodies (ANA, anti-SSA, anti-ds-DNA, aCL IgG, aCL IgM). Patients who had positive anti-ds-DNA and anti-SSA antibodies had lower serum 25(OH)D concentration, but this difference did not reach statistical significance.

Our study has several limitations. It was designed as a cross sectional study with only one measurement of serum 25(OH)D concentration. Study included patients who generally had a higher level of disease activity and it is difficult to transfer these results to patients with less active disease. Data on prevalence of vitamin D deficiency and insufficiency were not compared to demographically appropriate control group of healthy patients or with patients with other autoimmune rheumatic diseases. The advantages of our study is that vitamin D status was discussed from different aspects of the SLE, we examined relationship with different markers of disease activity (SLEDAI, anti-ds-DNA Ab) and influence of different medications. To our knowledge this is the first such study in patients with SLE in Serbia.

In conclusion, our study shows that vitamin D deficiency and insufficiency are common in patients with SLE. All of our patients had reduced serum 25(OH)D concentration: 32.6% had vitamin D deficiency, and 67.4% vitamin D insufficiency. There was no association of selected clinical manifestations and disease activity with vitamin D status. Patients who used vitamin D supplements did not have significantly higher 25(OH)D level. Further studies are needed in order to understand the role of vitamin D deficiency in pathogenesis and clinical course of SLE.
